# PREPARING THE NEXT GENERATION OF THE DEMENTIA CARE WORKFORCE VIA SERVICE LEARNING: A SCOPING REVIEW

**DOI:** 10.1093/geroni/igae098.3882

**Published:** 2024-12-31

**Authors:** Makenna Green, Jamie Mayer, Laura White, Gail Kouame

**Affiliations:** Northern Illinois University, Saint Charles, Illinois, United States; Northern Illinois University, DeKalb, Illinois, United States; University of Alabama at Birmingham, Birmingham, Alabama, United States; University of South Alabama

## Abstract

The prevalence of Alzheimer’s Disease and Related Dementia’s (ADRD) is rising rapidly with the aging of the baby boomer generation. A large workforce gap in dementia providers is widening with education as a frequently cited barrier to entry into the ADRD workforce (Mayer et al, 2023). This scoping review aimed to explore the extant literature on the use of service learning (SL) pedagogy related to ADRD in higher education settings for students in health-related majors. A systematic search was conducted using seven databases. Sources were included if participants were undergraduate/graduate students in any health-related discipline and if SL was utilized in conjunction with specific instructional paradigms (e.g., coursework, on-site workshops). SL, though individually defined by authors of included studies, was required to include community engagement with adults with ADRD; studies specific to clinical rotations were excluded. Included sources (n=32) were systematically extracted for characteristics including student demographics, SL components (learning instruction methods/training, experiences, reflection), and outcomes. Analysis of training revealed most ADRD SL paradigms relied on setting-specific training (n=15) over didactic academic instruction (n=7) or a combination (n=7). SL experiences ranged in type with informal activities (n=13) and creative arts (n=9) most commonly utilized to engage participants. The reflection component most commonly included open-ended reflective journals (n=15). Analysis revealed variability in how SL is represented in the literature. Despite this variability, sources frequently report SL as a viable option for educating the next generation of dementia service providers, with observed improvements in student attitudes and knowledge related to ADRD.

